# Application of flipped classroom combined with virtual simulation platform in clinical biochemistry practical course

**DOI:** 10.1186/s12909-023-04735-x

**Published:** 2023-10-16

**Authors:** Liangbo Sun, Dong Liu, Jiqin Lian, Mingzhen Yang

**Affiliations:** Department of Clinical Biochemistry, Army Medical University, No. 30, Gaotanyan Street, Shapingba District, Chongqing, 400038 Chongqing China

**Keywords:** Clinical biochemistry, Practical course, Virtual simulation platform, Flipped classroom, Icourse, DingTalk

## Abstract

**Background:**

The study explores an innovative teaching mode that integrates Icourse, DingTalk, and online experimental simulation platforms to provide online theoretical and experimental resources for clinical biochemistry practical courses. These platforms, combined with flipped classroom teaching, aim to increase student engagement and benefit in practical courses, ultimately improving the effectiveness of clinical biochemistry practical teaching.

**Methods:**

In a prospective cohort study, we examined the impact of integrating the Icourse and DingTalk platforms to provide theoretical knowledge resources and clinical cases to 48 medical laboratory science students from the 2019 and 2020 grades. Students were assigned to the experimental group using an overall sampling method, and had access to relevant videos through Icourse before and during class. Using a flipped classroom approach, students actively participated in the design, analysis, and discussion of the experimental technique. For the experimental operation part, students participated in virtual simulation experiments and actual experiments. Overall, the study aimed to evaluate students’ theoretical and operational performance after completing the practical course. To collect feedback, we distributed a questionnaire to students in the experimental group. For comparison, we included 42 students from the grades of 2017 and 2018 who received traditional instruction and were evaluated using standard textbooks as the control group.

**Results:**

The experimental group scored significantly higher than the control group on both the theoretical and experimental operational tests (82.45 ± 3.76 vs. 76.36 ± 3.96, P = 0.0126; 92.03 ± 1.62 vs. 81.67 ± 4.19, P < 0.001). The survey revealed that the experimental group preferred the teaching mode that combined the flipped classroom with the virtual simulation platform. This mixed method effectively promoted understanding of basic knowledge (93.8%, 45/48), operative skills (89.6%, 43/48), learning interest (87.5%, 42/48), clinical thinking (85.4%, 41/48), self-learning ability (91.7%, 44/48), and overall satisfaction compared with traditional methods (P < 0.05). This study demonstrates that an innovative teaching approach significantly improves the quality of clinical biochemistry practical courses and promotes students’ professional development and self-directed learning habits.

**Conclusion:**

Incorporating virtual simulation with flipped classrooms into clinical biochemistry practical teaching is an efficient and well-received alternative to traditional methods.

## Introduction

The rapid development of information network technology, computer technology, and internet technology has been integrated into various fields of society, providing new opportunities for transforming teaching modes in universities [1, 2]. Nowadays, the concept of educational informatization has gained wide recognition and attention. Most universities are gradually improving the construction of campus information-based teaching, integrating school teaching resources, and improving the teaching effectiveness of various disciplines [3, 4]. The adoption of teaching informatization can overcome the limitations of traditional teaching methods, such as the inability of students to attend offline classes in a timely manner and the constraints of limited teaching venues.

Clinical biochemistry, an important professional course in laboratory medicine, combines both theory and practice [5]. It is designed to provide the student with an understanding of the concepts of biochemical metabolism in normal and disease conditions and includes various techniques and methods for the detection and analysis of chemical components in body fluid samples. It also covers the principles of monitoring biochemical markers and the selection, establishment and evaluation of biochemical indicators for clinical use [6]. Clinical biochemistry practical courses typically comprise about 50% of the total curriculum and include both basic experimental principles and a significant number of hands-on procedures. These courses integrate clinical cases and incorporate the latest advances. As a result, practical training and clinical practice are an integral part of the overall teaching process for clinical biochemistry. The quality of the practical training environment has a direct impact on the students’ mastery of the subject. In the context of educational informatization, profound changes have occurred in laboratory medicine education and talent training models. The traditional classroom teaching mode for clinical biochemistry practical courses no longer meets the requirements of modern laboratory medicine talent cultivation. A single online or offline teaching mode cannot meet the evolving needs of discipline development and training a new generation of laboratory medicine talents. In summary, the exploration of hybrid online and offline teaching modes is a crucial area of focus in the current reform of laboratory medicine teaching.

Icourse, a learning management system operated and maintained by Higher Education Press Co., Ltd., provides students with convenient access to shared platform resources [7, 8]. We use Icourse, a high-quality online open course platform, as technical support for researchers. This, combined with offline interactive learning, allows us to enrich students’ learning methods and empower them to take the initiative in their learning. In addition, we use goal-oriented assignments to motivate students to complete the videos on the Icourse platform. Each course video includes a knowledge module with experimental indicators designed to enhance students’ ability to apply broad knowledge and foster innovative thinking. We maximize the use of the Icourse platform system to strengthen the supervision of the teaching process and continuously monitor the learning outcomes to ensure high quality teaching. Teachers use the Icourse platform for online teaching and also assign learning tasks before and after class, effectively integrating offline and online teaching methods. Another platform, DingTalk, is a free multi-terminal communication and collaboration platform that offers various features such as video conferencing, group discussions, posting assignments, and real-time recording and playback of courses [2, 9, 10]. Due to its powerful features, DingTalk has been widely adopted by major universities in China for online teaching. It eliminates time and space constraints in medical education and has proven effective in flipped classrooms. Both Icourse and DingTalk offer features such as class check-in, interactive classroom activities, video playback, supplemental learning, in-class testing and correction, and grade statistics and analysis to meet various forms of online teaching needs.

The flipped classroom teaching mode, supported by substantial evidence, is gaining popularity in higher education [11]. It is a reversal of the traditional classroom where teachers provide knowledge and students absorb it. In this model, students are encouraged to independently use the online learning resources in Icourse/DingTalk and virtual simulation platforms prior to class [12]. This allows them to access the resources at their own pace and convenience and make necessary modifications as needed. Then, under the guidance of the instructor, class time is devoted to collaborative and social application of the acquired knowledge. In an effort to improve the quality of teaching, we propose to integrate the flipped classroom approach into the teaching of clinical biochemistry laboratory courses and investigate the feasibility of combining the flipped classroom model with virtual simulation in practical laboratory sessions.

Virtual simulation technology is a three-dimensional virtual reconstruction of reality in a computer, providing users with an immersive experience of multiple senses such as sight, touch and hearing. It has a good degree of participation and operability of human-computer interaction to simulate a real laboratory learning environment [13, 14]. Various programs used in virtual laboratories can visualize concepts in theoretical courses, thereby promoting student learning. Virtual simulation has been widely used in medical education or training in basic, biomedical and clinical medicine [12, 15]. Many studies have shown that in undergraduate education, simulation-based learning experiences can better integrate theoretical knowledge with practice [16, 17]. Through repeated learning, learners can acquire the skills they need to react faster when working independently offline, and can better support experimental teaching. In addition, virtual simulation experiment teaching can break the space and time constraints of traditional experiments, providing users with a large number of experimental opportunities. If virtual simulation experiments are added to the teaching process of traditional practical courses, it can continuously strengthen students’ deeper understanding and memory of experimental theories and experimental operations, and can achieve better teaching results [18]. However, the effectiveness of virtual simulation in laboratory medicine teaching has not yet been evaluated, and whether online virtual experiments can achieve the expected teaching results has brought great challenges to practical teaching. In this study, the researchers aimed to evaluate the effectiveness of the teaching mode using flipped classroom combined with Icourse, DingTalk and virtual simulation experiments in clinical biochemistry laboratory teaching.

## Research methods

### Study design

Using a total sampling method, we selected a total of 48 students from all medical laboratory technology majors in the grades of 2019 and 2020. These students were designated as the experimental group for our prospective experimental study. The theoretical part of the practical course involved online learning before class through Icourse, and interactive discussions in flipped classrooms. To complement this, the experimental operation involved combining online virtual simulation experimental exercises with offline hands-on experimental operation exercises. To provide a basis for comparison, we also included a control group consisting of 42 students from all medical laboratory technology majors in 2017 and 2018. These students had completed traditional offline teaching of practical courses and experimental operation learning. The researchers retrospectively analyzed the theoretical and experimental results of both groups of students. It is important to note that all research participants had completed basic medical courses related to laboratory science and had some ability to apply medical knowledge for comprehensive analysis. The two groups of students were taught by the same teacher using the same curriculum and teaching materials. There were no significant differences in factors such as age, gender, and basic knowledge background among the study participants.

### Teaching context and methods

#### Experimental group

The practical course integrated online Icourse platform learning with face-to-face, flipped classroom interactions for a seamless blended learning experience. Students and teachers installed Icourse and DingTalk apps for easy collaboration. Four experienced teachers were assigned to both the Icourse class and DingTalk group for effective teaching. Twelve students and one teacher participated in the same Icourse class and DingTalk group, fostering collaborative learning and discussion.

In the theoretical component of the practical course, the teaching strategy entails the instructor’s selection of videos and typical clinical cases. These cases, based on instructional design, aim to address key clinical practice knowledge or challenging concepts. For instance, the instructor may select clinical cases like hyperlipidemia, focusing on important and difficult aspects of the curriculum, such as recognizing clinical biochemical indicators, understanding their clinical significance, performing biochemical differential diagnoses, and considering confounding factors in hyperlipidemia. This video-viewing and case study process was implemented in the classroom, lasting approximately 15 min. Relevant videos, theoretical content, clinical cases, and literature for each practical course were made available through the Icourse platform one week prior to class. Students were expected to learn independently before class. In class, students engage in autonomous learning by sharing relevant knowledge related to the case. They also organized and summarized questions posed by the teacher. In class, students shared relevant case knowledge, organize questions, and interact with the teacher. They designed experiments, analyzed designs, and thought deeply about clinical indicator detection. By introducing clinical cases, the course was aligned with real-world settings. The teacher led discussions were ensuring active participation. Post-discussion, feedback was provided, key points summarized, and hands-on activities proceed. This strategy enhanced subject comprehension and critical thinking skills.

**Fig. 1:**
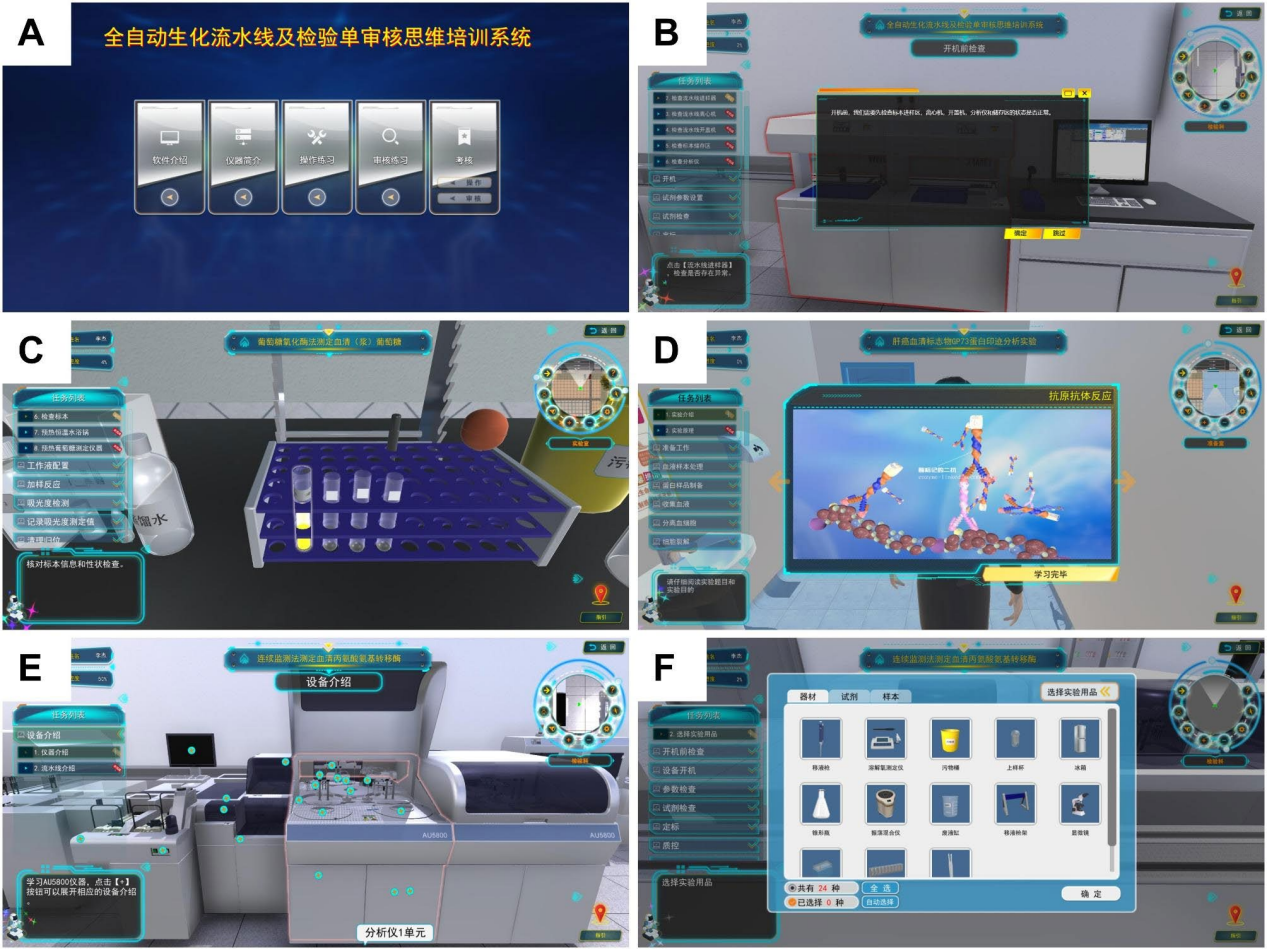
Virtual simulation laboratory component of the operation interface. **(A and B)** Automated biochemical analysis of virtual simulation systems. **(C)** Serum glucose was measured by oxidase method. **(D)** GP73 serum marker of liver cancer was detected by Western blot assay. **(E and F)** Serum alanine aminotransferase was measured by continuous monitoring method

The hands-on teaching strategy for clinical biochemistry experiments is indeed an innovative approach to enhance student learning. By using virtual simulation platforms, students could gain a deeper understanding of the experiments and their underlying principles. This method of teaching also allows students to practice and refine their skills outside of the classroom, which can lead to better retention of knowledge and skills. Moreover, the platform provided step-by-step interpretations, simulations, and other resources to support learning. This scaffolding was particularly helpful for students who may be struggling with the material, as it provided a structured way to learn and understand the concepts. The platform also allowed for different modes of engagement, such as demonstration mode and operation mode. This flexibility allowed students to learn at their own pace and according to their own learning style. In addition, through the virtual testing instruments, students could gain proficiency in understanding the internal structure, working principles, quality control, calibration, sample testing, reagent replacement, maintenance and alarm handling of automatic biochemical analyzers. An example of the partial user interface of the virtual simulation lab is shown in Fig. 1. Teachers used background sharing to monitor students’ operations, ensuring accuracy and standardization. Instructors viewed students’ work time, performance, and results via teacher accounts and address common errors committed during the process. After the virtual simulation, students conducted experiments based on the previously discussed experimental design.

#### Control group

In the theoretical part of the practical course, traditional teaching methods were used, where the teacher imparted the basic knowledge and theories of clinical biochemistry practical course according to the curriculum. During the practical sessions, the teacher explained the purpose, principles, methods, operational key points and precautions of the experiments. However, there was no integration of hands-on activities with virtual simulation platforms. During group experiments, the teacher moved around the classroom, providing guidance and assistance to the students. After completing the experiments, the students were required to write experiment reports according to the given guidelines. The teacher then evaluated and corrected the reports for assessment purposes.

### Student evaluation

Student evaluation in this study was divided into two parts. The first part was an evaluation of the effectiveness of the teaching after the end of the course. This included assessment of understanding of theoretical concepts and ability to perform experimental operations. The theory test assessed the students’ knowledge of basic theoretical concepts and their ability to analyze clinical cases. During the experimental operation, students were randomly assigned experimental tasks and their ability to complete these tasks accurately was evaluated. The test scores of the control group were obtained from the teaching materials for data analysis. In addition to the test scores, the study also includes the collection of students’ opinions through questionnaires. Three questionnaires were administered in this study. Questionnaire 1 and Questionnaire 2 aimed to investigate the students’ acceptance and perception of Icourse/DingTalk combined with virtual simulation experiment teaching. Questionnaire 3 focused on assessing the overall satisfaction of students in both the experimental and control groups with the teaching methods used. All participants in the study gave their informed consent prior to data collection.

### Statistical analysis

Statistical analysis was performed using SPSS 20.0 software (SPSS Inc., Chicago, IL). Continuous variables were presented as means and standard deviations, while frequencies and percentages were used to classify variables. Independent t-tests were used to analyze continuous variables that had a normal distribution, such as age and test scores. Chi-squared tests were used to analyze categorical variables such as gender (men/women) and satisfaction (satisfaction/ dissatisfaction). A p-value of less than 0.05 was considered statistically significant.

### Semi-structured interviews

In order to investigate the limitations of the teaching mode combining virtual simulation experiments and flipped classroom in clinical biochemistry teaching, we conducted semi-structured interviews with students who had completed the course. The steps are as follows: (1) Non-teaching staff members of our teaching team developed an interview outline, including overall experience of the virtual simulation combined flipped classroom model, experience of using the virtual simulation platform, experience and feelings of the flipped classroom model, as well as difficulties encountered in the teaching mode. (2) The aforementioned researchers randomly selected 13 students from the 48 students participating in the virtual simulation flipped classroom to ensure the sample was representative. (3) The researchers conducted face-to-face interviews, encouraging students to freely express their views and experiences, and guiding in-depth discussion through open-ended questions. Interview questions are as follows: What are the drawbacks of combining virtual simulation with the flipped classroom teaching model in your opinion? What are the negative experiences you have after using virtual simulation platforms? What are the weaknesses in the learning process of the flipped classroom model? What difficulties have you encountered in this teaching mode? (4) The two instructors used the Colaizzi analysis method [19, 20] to analyze the interview recordings and notes, extract key information, categorize, synthesize, and integrate the student feedback, in order to identify the main themes. (5) The researchers presented the survey results, including a detailed description, identification of similar viewpoints, and the thematic content of student feedback analyzed. (6) Researchers returned the analysis results to the respondents for verification, discussed with the research team, and determined the final coding and themes. Throughout the process, we strictly adhered to ethical principles to ensure the protection of students’ rights and interests, including obtaining informed consent from the respondents and keeping their personal information confidential.

## Results

### Course score comparison

A total of 90 students participated in the study, with 48 students in the experimental group and 42 students in the control group. Table 1 shows that there were no significant differences in age and gender between the two groups. At the end of the semester, the experimental group scored higher than the control group on the theoretical exam (82.45 ± 3.76 vs. 76.36 ± 3.96) and the practical skills exam (92.03 ± 1.62 vs. 81.67 ± 4.19). These differences were statistically significant as shown in Table 2.

**Table 1:** Basic information for two groups students

	Control group (n = 42)	Experimental group (n = 48)	P-value
Age	20.41 ± 0.74	20.1 ± 0.62	0.7528
Gender			0.6925
Men	36 (85.71%)	41 (85.42%)	
Women	6 (14.29%)	7 (14.58%)	

**Table 2:** Comparing the scores of two groups (the maximum score is 100)

	Control group (n = 42)	Experimental group (n = 48)	P-value
Practical course theory examination	76.36 ± 3.96	82.45 ± 3.76	0.0126
Practical course operation examination	81.67 ± 4.19	92.03 ± 1.62	< 0.0001

### A survey on the acceptance of the flipped classroom teaching method in the theoretical part of the practical course

At the end of the semester, a questionnaire (Questionnaire 1) was distributed to the students in the experimental group via Icourse to evaluate their acceptance of the Icourse-based teaching method in the theoretical part of the practical course. A total of 48 questionnaires were sent out and all 48 were returned, resulting in a valid response rate of 100% (Table 3). The results of the survey indicated that the flipped classroom teaching mode had positive results. The majority of the students (38 out of 48, 79.2%) preferred the flipped classroom teaching mode. They expressed a willingness to engage in classroom interactions with both peers and teachers (42 out of 48, 87.5%) and a desire to share their own ideas (44 out of 48, 91.7%). In addition, students found the teaching method valuable and interesting in terms of the quality of the clinical cases (42 out of 48, 87.5%). They also believed that it helped to improve their clinical skills (45 of 48, 93.8%). Moreover, the majority of students were satisfied with the teaching resources provided by the platform (47 out of 48, 97.9%). Students also rated other aspects of the teaching mode highly. They felt confident in their ability to complete assigned tasks on time (46 out of 48, 95.8%), apply prior knowledge to problem-solving scenarios (41 out of 48, 85.4%), and critically evaluate their acquired knowledge (42 out of 48, 87.5%). In addition, most students agreed that the instructors were able to provide timely feedback based on their questions on the Icourse and DingTalk platforms, thus enhancing their learning experience (45 out of 48, 93.8%). Finally, the majority of students (47 out of 48, 97.9%) believed that the flipped classroom teaching mode has the potential to be an effective tool for teaching the clinical biochemistry practical course.

**Table 3:** Questionnaire results of the teaching mode based on flipped classroom

Question	Yes n(%)	No n(%)
1. Do you like the teaching mode based on flipped classroom?	38 (79.2)	10(20.8)
2. Are you willing to actively interact with classmates or teachers in class?	42 (87.5)	6 (12.5)
3. Can you share your ideas as effectively as possible in class?	44 (91.7)	4 (8.3)
4. Do you think the clinical cases introduced in each Icourse are valuable and interesting?	42 (87.5)	6 (12.5)
5. Do you agree that this teaching method can improve clinical ability?	45 (93.8)	3 (6.2)
6. Are you satisfied with the teaching materials uploaded by your teacher in Icourse and DingTalk?	47 (97.9)	1 (2.1)
7. Can you complete the task on time?	46 (95.8)	2 (4.2)
8. Can you apply your learning to solve problems?	41 (85.4)	7 (14.6)
9. Can you critically evaluate the knowledge you have gained?	42 (87.5)	6 (12.5)
10. Do your team members and teachers provide timely feedback to enhance your learning?	45 (93.8)	3 (6.2)
11. Do you think the teaching mode based on flipped classroom can effectively improve the teaching effect of clinical biochemistry?	47 (97.9)	1 (2.1)

### An acceptance survey of the teaching mode based on virtual simulation experiments combined with flipped classroom

At the end of the semester, questionnaires were sent to the students in the experimental group (48 in total) through Icourse to evaluate their acceptance of the teaching mode. All 48 questionnaires were returned, resulting in a valid response rate of 100% (Table 4). The survey results indicate that the majority of students (95.8%) found the teaching mode, which included virtual simulation experiments and the use of Icourse, to be useful. In additionally, 85.4% of the students reported that they enjoyed this teaching method. A significant number of students (81.3%) felt that virtual simulation experiments provided a more realistic experience compared to traditional experiments. The ability to communicate with classmates and instructors through Icourse or DingTalk, and to receive feedback when encountering difficulties with virtual simulation experiments, was considered beneficial by most students (89.6%). The use of virtual simulation experiments made 89.6% of students feel more actively engaged in the learning process. The majority of students (97.9%) agreed that virtual simulation experiments improved their understanding of concepts, and 89.6% agreed that virtual exams were fair and objective. Furthermore, 81.3% of the students expressed their desire for further integration of virtual simulation experiments in future practical courses, as this would better prepare them for future internships (85.4%). Regarding the technical aspects, 97.9% of the students found the virtual laboratory easy to use. They reported that it was user-friendly and convenient to navigate, with clear instructions for each step (95.8%). The image and sound quality in the virtual laboratory was described as good by 97.9% of the students. However, a small number of students (6.3%) experienced slight discomfort such as dizziness while interacting with the virtual laboratory. To summarize, a significant majority of students (97.9%) believed that the teaching mode, incorporating Icourse/DingTalk and virtual simulation experiments, greatly contributed to the improvement of their theoretical knowledge and operative skills in the practical course.

**Table 4:** An acceptance survey of flipped classroom combined with virtual simulation experiments

Question	Yes n (%)	No n (%)
1. The virtual simulation laboratory platform is very useful for learning this discipline.	46 (95.8)	2 (4.2)
2. The combination of Icourse and virtual simulation experiment teaching method makes me happier.	41 (85.4)	7 (14.6)
3. Virtual laboratory courses provide a more realistic experience than traditional laboratory courses.	39 (81.3)	9 (18.8)
4. If I encounter problems during the operation process, I can communicate with teachers or classmates through Icourse/DingTalk in time.	43 (89.6)	5 (10.4)
5. I participate more actively if combined with the virtual laboratory.	43 (89.6)	5 (10.4)
6. Virtual laboratory helps me learn clinical biochemistry and increase my knowledge.	47 (97.9)	1 (2.1)
7. Virtual laboratory simulation tests are more impartial and objective than traditional experimental tests.	43 (89.6)	5 (10.4)
8. Compared to traditional laboratories, I prefer to use virtual laboratories to complete more testing projects.	39 (81.3)	9 (18.8)
9. I want to use this teaching method to prepare for my future internship.	41 (85.4)	7 (14.6)
10. The virtual simulation laboratory system is easy to use.	47 (97.9)	1 (2.1)
11. It is easy to log in the virtual laboratory by following the instructions provided.	46 (95.8)	2 (4.2)
12. It is easy for me to navigate in a virtual laboratory.	46 (95.8)	2 (4.2)
13. The video and audio quality of the virtual laboratory are very good.	47 (97.9)	1 (2.1)
14. I can comfortably look around the laboratory and conduct experimental operations without feeling any discomfort.	45 (93.8)	3 (6.3)
15.The combination of Icourse and virtual laboratory teaching has made me more confident in mastering knowledge and skills.	47 (97.9)	1 (2.1)

### Student evaluation

Upon completion of the study, questionnaires were distributed to students in both the experimental and control groups. A total of 90 questionnaires were returned, resulting in a 100% response rate. In Table 5, the percentage represents the perceived effectiveness of each teaching method among the respondents. This percentage indicated the level of student satisfaction with five specific learning outcomes. The survey results showed that the experimental group expressed higher levels of satisfaction compared to the control group in areas such as “understanding of basic knowledge”, “mastery of operational skills”, “development of learning interests”, “clinical thinking”, and “improvement of self-learning skills”. These differences were found to be statistically significant with a p-value of < 0.05, as shown in Table 5.

In addition to the questionnaires, semi-structured interviews were conducted with a subset of the participants to gain further insight into the limitations of the teaching mode in clinical biochemistry teaching. Thirteen students participated in these interviews, and their responses highlighted several reasons why they did not prefer this blended teaching method: (1) Time and energy requirements: The combination of Icourse/DingTalk with the virtual simulation platform required a significant amount of time and energy. In addition to regular courses and key discussions, students had to use their personal rest time to review a considerable amount of information. (2) Lack of realism in simulated scenes: Students felt that the simulated clinical laboratory scenes lacked realism compared to traditional laboratory teaching methods. Long online sessions also caused discomfort, such as feelings of loneliness and dizziness. (3) Network connectivity issues: Some students reported that their experience with the teaching mode was impacted by network connectivity issues, which hindered their ability to fully engage with the course materials. These findings shed light on the specific challenges students face with the teaching mode and provide valuable insights for improving future implementations.

**Table 5:** Comparison of satisfaction of teaching between two groups students

Question	control group %(n/42)	Experimental group %(n/48)	P-value
1. It helps to understand the basic knowledge.	73.8 (31/42)	93.8 (45/48)	0.0061
2. It helps to master operating skills.	78.6 (33/42)	89.6 (43/48)	0.0375
3. It helps to deepen learning interest.	66.7 (28/42)	87.5 (42/48)	0.0237
4. It helps to train clinical thinking.	73.8 (31/42)	85.4 (41/48)	0.0197
5. It helps to improve self-learning ability.	54.8 (23/42)	91.7 (44/48)	< 0.001

## Discussion

The conventional teaching approach in clinical biochemistry practical courses struggles to promote student engagement and develop critical thinking skills. In this model, the teacher is in a dominant position while the students are passive recipients of knowledge. This hinders students’ enthusiasm, motivation, and ability to learn, speculate, and collaborate independently [21]. Furthermore, traditional teaching methods focus on the accumulation of knowledge, with students simply following the teacher’s instructions when conducting experiments. This lack of communication and interaction between teachers and students makes it difficult to fully understand students’ learning progress and understanding of various concepts. As a result, students can easily forget experimental principles and procedures after the course ends. This approach also does not promote the development of independent learning and critical thinking skills, which hinders students’ overall personal growth. Overall, the traditional teaching mode in clinical biochemistry practical courses needs to be reconsidered in order to effectively engage students, foster critical thinking, and promote a holistic and personalized approach to learning [22].

The rapid development of computers and the Internet has had a profound impact on medical education [23]. Numerous medical institutions globally utilize online teaching platforms to augment their courses and facilitate learning. In 2018, China’s Ministry of Education launched the Education Informatization 2.0 Plan, aiming to foster the integration of education and the Internet. The COVID-19 pandemic and subsequent isolation measures further sped up the transition to online education [24–28]. Consequently, there’s a pressing demand for hybrid teaching methods fusing online and offline elements [29]. In this study, the teaching mode we designed could flexibly switch to a fully online format as needed to cope with unexpected situations, such as the lockdown period.

The integration of online independent learning and the flipped classroom is crucial in the current educational reform. Online learning improves resource utilization, overcomes location and time limitations, and allows teachers to focus on research rather than repetitive tasks [16]. Therefore, we effectively integrated face-to-face classroom teaching with online teaching, utilizing online resources to expand students’ learning opportunities and classroom time, to address complex topics, encourage discussions, and promote communication. Our results indicate that this teaching mode significantly improved students’ intrinsic learning motivation in clinical biochemistry practical courses, which is in line with the current trend of educational informatization [30, 31]. In implementing this model, face-to-face teaching in the flipped classroom is also essential for conducting more in-depth studies and discussions between teachers and students [32]. Therefore, we recommend implementing a blended learning approach in clinical biochemistry practical courses, combining online modules with offline case discussions to enhance students’ interest and understanding, create a student-centered learning environment, and improve teaching quality.

In recent years, there’s been growing interest in using online platforms like Icourse and DingTalk for medical education in China [8, 9, 33]. This study combined these platforms with traditional methods, creating a conducive learning environment that emphasizes independent learning and discussion. This approach combined the benefits of traditional teaching while overcoming time and space constraints. The virtual clinical biochemistry laboratory platform, accessible through a web browser, utilized VR technology and doesn’t require additional software installation [34]. The platform focused on practical training and skill improvement, featuring modules for virtual biochemistry, automated instruments, virtual experiments, and clinical reasoning. The teacher released learning tasks on the Icourse platform before the class, and students could preview the content of the next practical class, including methodology, principles, and clinical cases through the Icourse platform. Students could also communicate with teachers about any questions they had on DingTalk, which could help them better complete self-study before class. Meanwhile, teachers could track students’ learning data in the background of the Icourse platform. In the practical class, students watched related videos and engaged in discussions and interactions on clinical cases. They designed experimental plans for the projects they needed to test and performed learning operations on the virtual platform, understanding the principles of the detection equipment and the operation principles. After the virtual simulation, students conducted experiments based on the experimental plans they discussed earlier. Icourse and DingTalk offered a playback function for post-class review, encouraging students to explain answers and integrate prior knowledge. Icourse provided real-life clinical cases to stimulate problem-solving interest and integrate theory with practice. Students accessed top-notch course videos, enhancing their learning. These platforms facilitated post-class review, collaboration on challenges/projects. This multi-channel approach boosted class interaction and student collaboration.

Traditional clinical biochemistry experimental teaching methods have certain limitations, such as limited equipment and space, outdated content, and large gaps from actual clinical techniques. Virtual simulation experiments have many advantages, such as the ability to conduct experiments in various environments, wide content coverage, video teaching, standardized instruments and procedures, and access at any time and place [35]. In our teaching mode, students could practice virtual simulation experiments before and after class, which can improve their understanding and application of experimental principles and operations [12, 36]. In this specific study, students who received virtual simulation experiment guidance performed significantly better than the control group. This is because the difficulty in mastering experimental skills lies in memorizing and understanding standardized steps and procedures, and virtual simulation experiments can fully replicate these steps and procedures. Therefore, the implementation of virtual simulation experiments has a positive impact on the learning outcomes of students in clinical biochemistry practical courses.

Applying motivation theory to education can improve students’ enthusiasm and teaching effectiveness [37]. Understanding students’ motivational needs helps to design strategies based on self-determination [38], achievement motivation [39], and expectancy theory [40], which enhance learning motivation and outcomes. Firstly, self-determination theory emphasizes autonomy and personal choice of actions [41]. Therefore, providing course design options can stimulate students’ interest and initiative, and strengthen learning motivation. Secondly, achievement motivation theory believes that students’ motivation and behavior are driven by their desire for success and achievement. We enhanced students’ autonomous learning motivation by setting challenging goals before class, and promptly acknowledging and appreciating their efforts. This approach improved students’ self-confidence and practical skills, thereby encouraging them to actively participate in practical courses. Thirdly, expectancy theory indicates that individuals’ motivation is influenced by their expectations and values regarding future outcomes. We could emphasize the importance of these courses to their future career development and the value of applying practical skills in real life to enhance students’ motivation for learning practical courses. In clinical biochemistry practical courses, we could effectively improve students’ motivation for autonomous learning by applying motivation theory. This could be achieved by providing choices, setting challenging goals, and emphasizing the importance and application value of practical courses.

The effectiveness of hybrid teaching, which combines virtual simulation platforms, online teaching tools such as Icourse/DingTalk, and the flipped classroom, in improving student performance is a complex issue. It is influenced by various factors, including student preparation, course content, teaching strategies, and evaluation methods. The shift from passive to active learning in the online teaching requires students to change their attitudes and behaviors, which can be challenging for those accustomed to traditional teaching methods. Teachers need to be aware of the potential setbacks students may experience in self-directed online learning [42, 43]. Another concern is the lack of evaluation of differences in teaching quality among different teachers, despite the use of the same curriculum and materials. In addition, there may be differences in the academic level of students enrolled in the same major but in different academic years. Reflections on the teaching mode of Icourse/DingTalk combined with virtual simulation experiments include the need to optimize online course resources and diversify the available online courses. Coordination between online and offline content and scheduling should also be carefully managed to minimize the impact on students’ free time. It is important to improve the organization and consolidation of various experimental knowledge points and to establish stronger links between chapters. Furthermore, integrating case-based teaching throughout the theoretical teaching process can effectively bridge the gap between theoretical knowledge and clinical practice.

### Limitations

In this study, we explored the application of hybrid teaching in a clinical biochemistry practice course. This approach combined the use of virtual simulation platforms, online teaching tools such as Icourse/DingTalk, and the flipped classroom. However, it is important to acknowledge the following limitations of our study. First, the transition from passive to active learning requires students to change their attitudes and behaviors. Therefore, the challenge lies in improving students’ learning motivation and initiative. It is crucial for teachers to address potential setbacks that students may encounter during autonomous online learning. Second, students’ engagement in autonomous learning before class may consume their free time. Therefore, it is necessary for teachers to carefully reconstruct the curriculum in a scientifically designed way to minimize the impact on students’ leisure time. Third, the simulation scenes may lack realism, and occasional system errors may undermine students’ sense of learning experience. Thus, continuous optimization and improvement of online platform resources is imperative. In terms of questionnaire design, we have chosen to include only “yes/no” response options for specific purposes and research needs. However, it is important to recognize that this limited response format may limit the richness of the data collected and may not allow for more detailed or varied responses. To overcome this limitation and gather more in-depth information, it may be necessary to include additional types of scales or open-ended questions at later stages of the survey.

## Conclusions

In conclusion, Icourse and DingTalk are popular online learning platforms and instant messaging applications in China that are widely used in teaching clinical biochemistry. These platforms create an interactive learning environment that facilitates student engagement. The integration of online and offline flipped classroom teaching through Icourse/DingTalk improves students’ understanding of theoretical knowledge in practical courses. In the experimental operation section, onsite experimental activities are combined with virtual simulation experiments to enhance students’ skill acquisition. This teaching mode has been validated as an effective approach in experimental medicine education. In particular, it can serve as a potential solution for acute infectious disease outbreaks and the transition to online teaching. Additionally, it introduces new teaching strategies that can be applied to other clinical disciplines.

## Data Availability

The datasets analyzed during the current study are available from the corresponding author on reasonable request.
